# Glial cell deficits are a key feature of schizophrenia: implications for neuronal circuit maintenance and histological differentiation from classical neurodegeneration

**DOI:** 10.1038/s41380-024-02861-6

**Published:** 2024-12-05

**Authors:** Hans-Gert Bernstein, Madeleine Nussbaumer, Veronika Vasilevska, Henrik Dobrowolny, Thomas Nickl-Jockschat, Paul C. Guest, Johann Steiner

**Affiliations:** 1 Department of Psychiatry, Otto-von-Guericke-University Magdeburg, Magdeburg, Germany; 2 Laboratory of Translational Psychiatry, Otto-von-Guericke-University Magdeburg, Magdeburg, Germany; 3 Department of Radiotherapy, Otto-von-Guericke-University Magdeburg, Magdeburg, Germany; 4 Department of Neuroscience and Pharmacology, Carver College of Medicine, University of Iowa, Iowa, IA USA; 5 Iowa Neuroscience Institute, University of Iowa, Iowa, IA USA; 6 Department of Psychiatry, Carver College of Medicine, University of Iowa, Iowa, IA USA; 7 Center for Behavioral Brain Sciences (CBBS), Magdeburg, Germany; 8 German Center for Mental Health (DZPG), Partner Site Halle-Jena-Magdeburg, Magdeburg, Germany; 9 Laboratory of Neuroproteomics, Department of Biochemistry and Tissue Biology, Institute of Biology, University of Campinas (UNICAMP), Campinas, Brazil

**Keywords:** Neuroscience, Schizophrenia

## Abstract

Dysfunctional glial cells play a pre-eminent role in schizophrenia pathophysiology. Post-mortem studies have provided evidence for significantly decreased glial cell numbers in different brain regions of individuals with schizophrenia. Reduced glial cell numbers are most pronounced in oligodendroglia, but reduced astrocyte cell densities have also been reported. This review highlights that oligo- and astroglial deficits are a key histopathological feature in schizophrenia, distinct from typical changes seen in neurodegenerative disorders. Significant deficits of oligodendrocytes in schizophrenia may arise in two ways: (i) demise of mature functionally compromised oligodendrocytes; and (ii) lack of mature oligodendrocytes due to failed maturation of progenitor cells. We also analyse in detail the controversy regarding deficits of astrocytes. Regardless of their origin, glial cell deficits have several pathophysiological consequences. Among these, myelination deficits due to a reduced number of oligodendrocytes may be the most important factor, resulting in the disconnectivity between neurons and different brain regions observed in schizophrenia. When glial cells die, it appears to be through degeneration, a process which is basically reversible. Thus, therapeutic interventions that (i) help rescue glial cells (ii) or improve their maturation might be a viable option. Since antipsychotic treatment alone does not seem to prevent glial cell loss or maturation deficits, there is intense search for new therapeutic options. Current proposals range from the application of antidepressants and other chemical agents as well as physical exercise to engrafting healthy glial cells into brains of schizophrenia patients.

## Introduction

Schizophrenia (SCZ) is a serious brain disorder that affects approximately 24 million people, or 1 in 300 people, worldwide [1]. The disease is mostly regarded as a neurodevelopmental disorder involving changes in brain circuitry that progress throughout life [2–4]. The aetiology of SCZ is still not completely understood, but appears to emerge from a complex interplay of polygenic risk and environmental factors [5]. After decades of research into the neurobiological basis of SCZ, it is becoming increasingly apparent that aberrant glial function is a major contributor to the pathophysiology of this disease [6–11].

Normal brain development involves neuronal proliferation, migration, synapse formation, arborisation (circuit formation) and myelination [4]. The first two processes mostly occur prenatally, while the latter two are crucial for brain maturation into early adulthood [4, 12]. The prefrontal cortex is the last  brain region to mature, which involves the pruning of redundant synapses, synaptic plasticity and increased myelination throughout adolescence and early adulthood, during the period of prodrome and onset of psychosis [4, 12]. Glial cells play a crucial role in this process. Excessive synaptic pruning by microglial cells (MCs), reduced myelination and energy supply of axons by oligodendrocytes (OLs), and impaired synaptic plasticity, synaptic glutamate recycling and neuronal energy supply due to astrocyte (AC) dysfunction may lead to an excitatory-inhibitory imbalance and increased vulnerability (e.g. to psychosocial trauma/stress, illicit drugs, infections, autoimmunity) of prefrontal brain circuits at this stage of life (Table 1) [13–17].

**Table 1:** The role of glial cells in the formation and maintenance of neuronal circuits.

Glial cell type	Role in neural circuit formation and maintenance
Oligodendrocytes (OLs)	1. Myelination and Energy Supply: Parafascicular OLs myelinate axons, ensuring proper speed and timing of action potentials essential for synaptic strength and plasticity [45, 83, 84].
	2. Neurotrophic Support: Perineuronal OLs release neurotrophic factors and provide metabolic support, influencing neuronal health and plasticity [43].
	3. Environmental Maintenance: Pericapillary OLs contribute to maintaining the neural environment and blood-brain barrier integrity, indirectly supporting synaptic plasticity [85].
Astrocytes (ACs)	1. Synaptic Plasticity: ACs regulate synaptic plasticity, crucial for learning and memory, by modulating neurotransmitter levels and synaptic strength [16].
	2. Glutamate Recycling: ACs are responsible for recycling synaptic glutamate, preventing excitotoxicity and supporting neuronal health [15].
	3. Energy Supply: ACs provide metabolic support to neurons, delivering essential nutrients and maintaining energy homeostasis [15].
Microglia Cells (MCs)	1. Synaptic Pruning: MCs are involved in synaptic pruning, removing redundant or weak synapses to refine neural circuits and enhance synaptic efficiency [14].
	2. Immune Surveillance: MCs act as the brain’s immune cells, identifying and responding to pathogens or injury, thereby protecting neural circuits [17].
	3. Inflammatory Response: Upon activation, MCs can release inflammatory mediators that influence neuronal health and synaptic function, potentially contributing to neuropsychiatric disorders if dysregulated [17].

Glial cell loss is a key feature of SCZ histopathology. Post-mortem findings replicated by several laboratories have provided evidence for significantly reduced glial cell densities in different brain regions of individuals with SCZ [6, 7, 18–23]. Notably, these changes have not only been observed in prefrontal brain, but prefrontal circuits are particularly sensitive during the typical age of onset of  SCZ. Although the phenomenon of decreased glial cell densities is most pronounced in OLs, it is not restricted to this glial cell type, since reduced AC densities have also been reported [18, 24–29]. While MCs have also attracted increasing attention recently [30, 31], our review focuses exclusively on OLs and ACs for the sake of clarity and consistency.

In search of further explanations for OL deficits in SCZ, the development of these cells has been analysed [32–35]. The identification of developmental irregularities led to the hypothesis that a major reason for OL deficits might be a disruption in their maturation [32, 35–40]. If so, we should have to reconsider certain aspects of glial cell loss in SCZ concerning the sources, pathophysiological consequences and possible ways to prevent cell loss.

The present review aims at increasing our understanding of how lower cell numbers of OLs occur as part of the pathophysiology of SCZ. This could indicate either that OLs are being lost or that they are less because their formation or maturation is reduced by neurodevelopmental disruption. These considerations will be discussed in the broader context of AC and OL abnormalities. Finally, we will highlight potential novel therapeutic approaches targeting glial cell deficits in SCZ, as a means of slowing the progression of this disease and alleviating symptoms.

## Search strategy

This review is based on a literature search including doctoral theses and patents in PubMed and Google Scholar databases from 1980 to 18 May 2024. The search terms were “schizophrenia” in combination with one or more of the following terms: “glia”, “gliosis”, “astrocytes”, “oligodendrocytes”, “oligodendrocyte progenitor”, “microglia”, “network disconnectivity”, “histopathology”, “glial cell loss”, “glial cell deficits”, “dysfunctional glia”, “myelin”, “protein expression”, “RNA expression”, “morphology”, “glial cell counting”, “GFAP”, “glial cell differentiation”, “glial cell maturation”, “atrophy”, “degeneration”, “necroptosis”, “apoptosis”, “phagocytosis”, “glial cell repair”, “medication”, “antipsychotic”, “neuroleptic”, “pathophysiological significance”, “therapy”, “aerobic exercise”, “glial cell transplantation”, “animal model”, and “cell culture”. No language restrictions were applied.

## Oligodendrocytes (Ols)

### Oligodendrogliopathy and myelin loss are core features of SCZ pathology

Although there has been some discrepancies in reporting, it is now assumed that the ratio between glia (ACs, OLs, MCs) and neurons in normal human brains is around 1:1 [41], and most reports state that OLs are the most abundant glial cell [42]. Based on localisation, Pío del Río Hortega subdivided OLs into four subtypes: (i) cells arranged in rows between nerve fibres, (ii) vascular satellite cells, (iii) Schwannoid OLs, and (iv) cells as satellites to neurons, juxtaposing neuronal perikarya [43, 44].

For decades, the importance of OLs for normal brain functioning has been underestimated. The main task of these cells is the proper myelination of many, but not all, neurites in the developing and adult brain, which is critical for rapid axonal conductance, correct information flow between neurons and metabolic supply [45]. Various lines of evidence converge to implicate OL and myelin deficits in SCZ. It has been shown that impairments in myelination of fibre tracts can lead to reduced white matter integrity in SCZ [46, 47]. This may lead to disturbed network connectivity, regarded as a hallmark of the disease [48, 49].

Histological and molecular profiling studies have revealed structural disarrangement and defects of the chemical makeup of myelin in SCZ. Myelin water imaging studies support evidence of abnormalities in white matter of people with SCZ [50]. Using light microscopy, less intense staining of deep white matter myelin has been observed in SCZ compared with controls [51]. At the electron microscopic level, a distorted organisation of myelin sheath lamellae has been found, which is characterised by reduced compactness, formation of concentric lamellar bodies, and inclusions between lamellae [52–54]. The demyelinating quaking mouse model of SCZ involves mice with a mutation in the QKI gene, leading to myelin deficiencies and SCZ-like symptoms. Remarkably, the myelin pathology found is nearly identical to what has been observed in post-mortem brains of individuals with SCZ [55]. This model links myelin abnormalities to SCZ, highlighting the importance of genes involved in myelin production and the impact of disrupted neural connectivity. There is also good evidence for multiple changes in the chemical composition of myelin [56, 57]. Proteomic and gene expression analyses have identified decreased concentrations of myelin-associated glycoprotein (MAG), quaking 1, 2´, 3´-cyclic nucleotide 3´-phosphodiesterase (CNP) messenger Ribonucleic Acid (mRNA) transcripts, transferrin, phospholipids and other components in white matter in SCZ [8, 56, 57]. Moreover, a subset of myelin-related genes has been implicated in SCZ by genetic linkage analysis [58, 59].

**Fig. 1:**
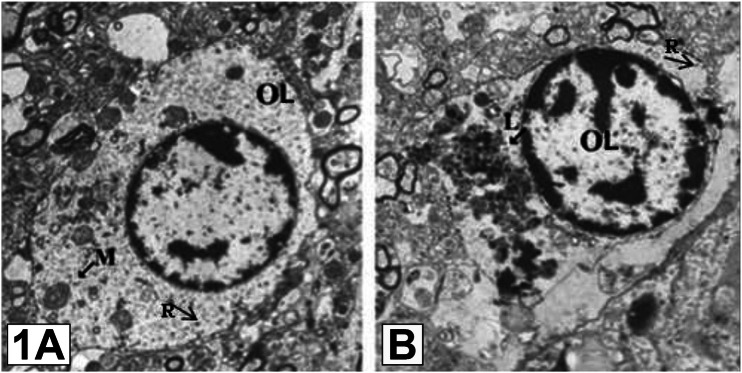
Electron microscopy of prefrontal white matter shows a normal OL in a healthy control subject (panel A) in comparison to a dystrophic OL in a schizophrenia patient (panel B). M Mitochondria, OL Oligodendrocyte, L Lipofuscin. R ribosomes (arrows); Scale bar = 2 μm. Reproduced with permission from Elsevier [73].

These myelin abnormalities have raised suspicions that the myelin-producing OLs themselves, including their development and function, are at least partly disrupted in SCZ [32]. Robust gene-expression evidence has also identified SCZ-associated alterations in OL profiles. Using a large SCZ genome-wide association study (GWAS), Goudriaan et al. found 29 OL genes strongly associated with SCZ, and the majority of these coded for myelin-shaping proteins [58]. Microarray, proteomic and immunocytochemical studies have shown that most OL proteins show decreased abundance in SCZ. Amongst these were CNP, MAG, erb-b2 receptor tyrosine kinase 3 (ErbB3), Oligodendrocyte transcription factor 2 (OLIG2), A disintegrin and metalloproteinase domain-containing protein 12 (ADAM12) and Neurite outgrowth inhibitor B (Nogo B) [60–66]. Only some were found to be up-regulated, including Neuregulin 1 (NRG1), erb-b2 receptor tyrosine kinase 4 (ErbB4), S100B, Neurite outgrowth inhibitor C (Nogo C), Disrupted In Schizophrenia 1 (DISC1) and prohibitin [66–70]. Uranova et al. showed that OLs from SCZ individuals had pronounced pathomorphological alterations (Fig.1). Follow up investigations found that OLs in various cortical and subcortical areas of the SCZ patients in this group had dystrophic and degenerative changes in structure. This sometimes featured swelling, reduction in the area of the nucleus and volume density of chromatin, and changes in the cytoplasm and cellular organelles, including reduction of the number of mitochondria and ribosomes, and accumulation of lipofuscin granules [52, 54, 71–73].

### Reduced number of oligodendrocytes in schizophrenia

Findings of reduced OL numbers in white and grey matter areas have been replicated in histological studies of SCZ, mostly by morphometric estimation of cell numbers on Nissl-stained sections [7, 19–23, 54, 71–76] (although two studies did not find this [77, 78]). In studies, which reported losses, reductions were approximately 30% in certain brain regions [54, 73, 74]. Decreased OL densities were also found when applying CNP staining to detect mature OLs [74]. Interestingly, illness-associated OL losses were not restricted to parafascicular OLs as the main myelin producers, but also involved cortical perineuronal and pericapillary OLs [79–82]. All these OL subtypes are involved in synaptic plasticity through various mechanisms (Table 1): 1.) Parafascicular OLs are responsible for the myelination and energy supply of axons, modulating the speed and timing of action potentials, which is essential for synaptic strength and plasticity [45, 83, 84]. 2.) perineuronal OLs release neurotrophic factors and provide metabolic support that affects neuronal health and plasticity [43], and 3.) pericapillary OLs contribute to maintaining the neural environment and blood-brain barrier integrity, indirectly supporting synaptic plasticity [85].

Death of mature OLs is one possible reason for their reduced numbers in SCZ. Given the observed ultrastructural cellular dystrophic and degenerative changes of OLs in SCZ, *degeneration with subsequent cell death* could be a cause of this [81]. Findings of smaller OL size and reduced function may be a consequence of *cell atrophy* – a pathological process characterized by a reduction in cell size and function due to a loss of cell substance. Atrophic cells generally become smaller and their metabolic activity and functionality are significantly impaired, although they remain viable. This process is analogous to the proposed fate of astrocytes (ACs) in SCZ as discussed in the AC chapter [86–89]. Of note, both atrophy and degeneration are reversible in their early stages. Another possibility was suggested by Hu and colleagues who showed that sustained ErbB receptor activation in *Plp*-tTA (Proteolipid Protein promoter driving expression of the tetracycline transactivator) transgenic mice caused inflammatory demyelination and hypomyelination through necroptosis of mature OLs [33]. This may be relevant as the observed white matter abnormalities were reminiscent of histopathological characteristics found in SCZ brains, and it is known that in SCZ neuregulin-ErbB signalling in OLs is disturbed [90].

*Necroptosis*, another pathological process, is a type of regulated cell death triggered by death receptors and a pathological hallmark in multiple sclerosis [7, 91]. Whether or not necroptosis is relevant for OLs losses in SCZ remains to be established, although cell death of mature OLs is different from that of differentiating OLs. Importantly, not all OLs may be affected by the aforementioned pathological processes. Instead, dysfunctional OLs, some of which undergo degeneration, atrophy or necroptosis [7, 33, 86–89], can coexist with unaffected ones [32].

Recent findings show that cuprizone treatment (a standard approach to study mechanisms of OL death) initiated a caspase-3–dependent form of rapid cell death only in differentiating OLs, while mature OLs did not activate this pathway and exhibited delayed cell death [92]. It is also known that during normal brain development, MCs digest OL progenitor cells [93], raising the possibility that activated MCs might also phagocytose mature OLs in SCZ. However, Uranova et al. found no evidence for increased OL dystrophy in association with MC activation [54].

Theoretically, OL deficits may be a consequence of medication, since clozapine and haloperidol treatments have been shown to cause multiple molecular alterations in human cultured OLs [94, 95]. Moreover, antipsychotic treatment has been shown to enhance mitochondrial autophagy in OLs [96]. However, OL numbers were not found to be significantly reduced in macaque monkey brains after chronic exposure to antipsychotics [97]. Moreover, quetiapine was shown to enhance OL regeneration and myelin repair in the cuprizone-induced demyelination mouse model of multiple sclerosis and SCZ [98]. Thus, medication is unlikely to initiate OL death.

With regard to pathophysiological consequences of OL losses, demise of a single OL can lead to loss of myelin sheath for several internodes of 20-60 axons, amplifying the loss to multiple neurons [34]. This will disrupt axonal conductance and metabolic supply of the affected fibres. Thus, massive OL losses will leave high numbers of neurites either partially or completely unmyelinated, contributing to the well-documented, disturbed white matter integrity and network disconnectivity in SCZ [46]. This network disconnectivity might have effects on multiple brain functions, including higher order brain processes [99]. Based on the results of their stereological study, Falkai et al. proposed that the decreased number of OLs in the hippocampus may contribute to cognitive deficits in SCZ by impairing the connectivity of this brain structure [37]. Another stereological study identified reduced OL numbers in the prefrontal cortex [75]. This could also lead to negative effects on glutamate [100] and dopamine functions [101, 102].

### Oligodendrocyte Progenitor Cells (OPCs)

Another potential cause for lower mature OL numbers in SCZ could be a diminished formation or maturation of OPCs. Indeed, a decrease in the numerical density of clusters containing OPCs has been recently reported in SCZ [23]. OLs are generated from OPCs, which emerge from radial glia during development [40]. These continuously self-renewing cells constitute 5–8% of the total cell population in the central nervous system (CNS) [103]. In humans OL development starts at the beginning of the second trimester and continues into adulthood [104]. The first signs of myelination can be found in the fetal human brain gestational months 3-4 [105]. During their development human OPCs undergo successive symmetric divisions to exponentially increase the progenitor cell pool size [32, 106]. The steps of OL development can be characterised according to their migratory capacity, increase in morphologic complexity and expression of specific cell markers [107]. OL maturation can be divided into four main stages: OPC, pre-OL (or late OPC), immature (or pre-myelinating) OL and mature (or myelinating) OL [107]. Typical specific stage markers are platelet-derived growth factor receptor α, the proteoglycan NG2 (both expressed in OPCs [108]) and G-protein receptor (GPR)17 (expressed in pre-OLs [109]).

### Emerging roles for reduced OPC numbers in SCZ

According to recent publications, many OL deficits in SCZ might result from failed OL maturation [32, 35–40, 110, 111]. This hypothesis assumes failure in some aspect of OPC differentiation in SCZ [34, 112]. Katsel and co-workers were the first to identify OL cell cycle abnormalities in SCZ [34]. The results of their microarray and quantitative real time PCR analyses provided evidence for gene expression changes of key regulators of G1/S cell cycle phase transition and genes central to OL differentiation in SCZ subjects. Their findings point to abnormal cell cycle re-entry in post-mitotic OLs. The same group also showed that OL and myelin deficits in SCZ involved failure of OPCs to exit the cell cycle for differentiation and maturation into OLs [76].

**Fig. 2:**
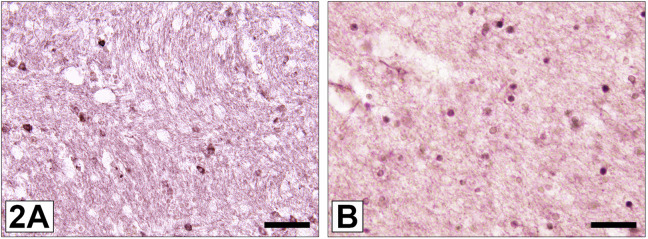
Immunohistochemical localisation of two OL markers, for which altered expression patterns in SCZ have been described in the literature. **A** White matter DISC1-immunoreactive OLs. The density of DISC1-expressing OLs was found to be enhanced in SCZ [70]. DISC1 negatively regulates differentiation of OPCs into OLs [38, 110, 116, 117]. **B** White matter OLIG2 expressing OLs. The density of OLIG2 immunopositive OLs was found to be reduced in SCZ [35, 82]. OLIG2 promotes OL differentiation [118].

Other studies have provided further clues for possible cell cycle abnormalities in SCZ. Leucine rich repeat and Immunoglobin-like domain-containing protein 1 (LINGO-1), an identified negative regulator of OPC differentiation and myelination [32, 113] was found to be present at higher levels in brains of subjects with SCZ compared to non-psychiatric controls [114]. Also, we demonstrated increased expression of the cell cycle regulator protein prohibitin in white matter OLs in SCZ [69, 115]. Moreover, an increased expression of DISC1 in mature OLs (Fig. 2A) and enhanced expression of the DISC1-Δ3 variant in OPCs have been identified in SCZ [70, 110]. DISC1 is known to negatively regulate differentiation of OPCs into OLs [38, 110, 116, 117]. Furthermore, some but not all investigators found reduced expression of OLIG2, which promotes OL differentiation [118], in brains of SCZ patients (Fig. 2B) [35, 82, 110]. In addition, stress-induced immune activation of MC during development appears to contribute to defective differentiation competence of OPCs [9, 38, 111].

Syed and co-workers showed that differentiation of cultured OPCs is inhibited by certain myelin proteins [119]. Theoretically, such an interaction may also take place in SCZ and illness-associated partial disintegration of myelin can lead to release of proteins that affect OPC function [52]. To investigate the proposed dysregulation of OL differentiation in SCZ, Mauney et al. compared the density of NG2- and OLIG2-immunoreactive cells between SCZ and normal control subjects [35]. They found that the number of cells expressing the specific late OPC marker NG2 was unchanged in SCZ, and concluded that late OPCs were numerically unaltered. That would mean that, despite potential developmental impairments, OPCs survived up to this stage but still needed to undergo maturation to myelinating OLs.

**Fig. 3:**
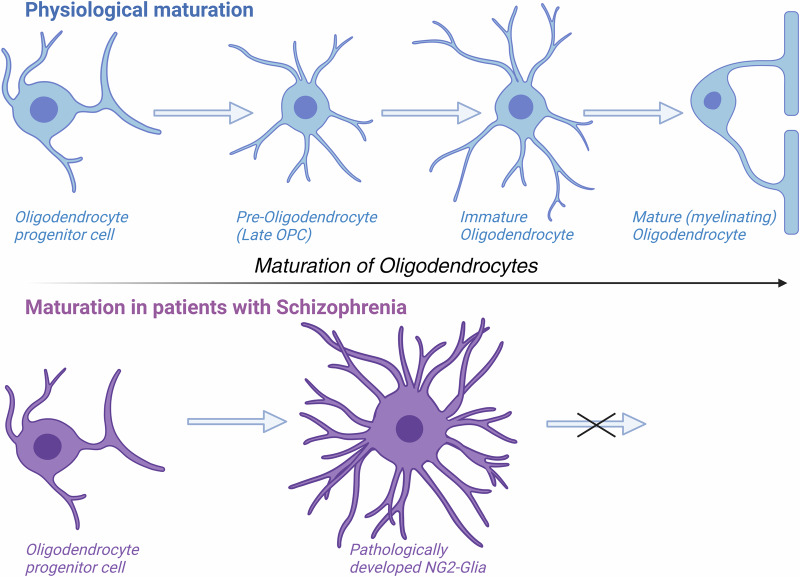
Normal maturation of human OLs (upper row) and disturbed OL maturation in SCZ (lower row). Schematic drawing based on the papers of Barateiro and Fernandes [107], Manuney et al. [35] and Yu et al. [110]. Figure generated with Biorender (www.biorender.com).

Unaltered numbers of NG2-expressing OPCs in SCZ were recently confirmed by other researchers [110]. However, it was also shown that these cells have an abnormal morphology, with hypertrophy, an elevated number of branches and greater branch lengths in SCZ patients [110]. Such morphological alterations might contribute to their inability to finish development **(**Fig. 3**)**. However, under normal conditions only some of the NG2-expressing cells differentiate into mature OLs [120]. Second, it must be taken into account that in SCZ a significant portion of NG2 expressing cells successfully transform into myelinating OLs. This provides an explanation for the coexistence of “healthy” (myelin-generating mature OLs) and “sick” (non-generating mature OLs) [32]. However, some findings suggest OPC losses occur at earlier stages of differentiation. In multiple brain regions, studies have found significant reductions in density of OL clusters, which are typically composted of 2–9 OPCs at different stages of development [23, 121, 122]. This is in agreement with earlier observations of Hof et al. which found that spatial distribution of OLs exhibited a “less clustered arrangement” in SCZ [75]. Of note, analysis of Nissl-stained sections showed that immature OLs are spatially organised both within and outside clusters and are morphologically indistinguishable from mature OLs [81]. Thus, it cannot be excluded that OLs and OPCs were counted together in SCZ studies using this staining method. Thus, it is not clear to which extent losses of OPCs and/or of mature OLs, contribute to the reduced number of these cells in SCZ [36].

Using an animal model of multiple sclerosis (*Plp*-tTA transgenic mice), Hu et al. showed that long term over-activation of the ErbB receptor causes demyelination by driving necroptosis of mature OLs and apoptosis of OPCs [33]. However, as mentioned before, it is not known if results of such model experiments reflect the situation in SCZ. Concerning the influence of medication on OL differentiation and maturation, we are not aware of any papers which reported OL developmental abnormalities in drug-naïve patients with SCZ. Hence it cannot be said, if antipsychotic treatment initiates and/or advances disruption of OL maturation. Instead, most communications deal with positive effects of antipsychotics on OL development.

Antipsychotics have been shown to promote differentiation of OPCs by regulating the transcription factors OLIG1 and OLIG2 [118]. A study of the hypothalamus of olanzapine-treated mice identified an increase in numbers of new-born cells differentiating to the OL lineage but not the neuronal lineage [123]. The same research team showed that olanzapine stimulated proliferation but inhibited differentiation of cultured rat OL progenitors [124]. Haloperidol is capable of activating quiescent OPCs in the adult mouse brain [125], while quetiapine was found to modulate the OPC cell cycle and thereby facilitate OL differentiation [126, 127]. Finally, we described protective effects of haloperidol and clozapine on energy-deprived Oligodendrocyte Line 93 (OLN-93) OLs [128]. This is of relevance because metabolic and oxidative stress are known to impair OPC differentiation in some brain disorders including SCZ [129, 130].

The main pathophysiological outcome of a deficit of mature OLs in SCZ is hypo-myelination with the aforementioned negative consequences. However, there is evidence that OPCs also have important non-myelinating functions. For instance, they possess an immunomodulatory capacity [46] and are involved in regulation of interneuron migration [103]. The latter property is of particular interest because disturbed OPC differentiation and migration in SCZ might contribute to abnormalities in connectivity proposed to take place in the disease [131]. Various aspects of glial cell losses in SCZ are summarised in Table 2.

**Table 2:** Loss of oligo- and astroglial cells in SCZ. A comparison between OLs, OPCs and ACs.

Sources, impact on pathophysiology, and therapy options	OLs	OPCs	ACs
Presence of glial cell deficits	Replicated findings from different laboratories point to prominent cell losses in different brain areas [7, 19–23, 71–76, 79–82].	Deficits arising from failure of OPCs to become myelinating OLs and/or deficits from loss of differentiating OPCs (reduced number of clusters containing differentiating OPCs) [23, 35, 72].	Controversially discussed. A number of GFAP based studies showed mild cell losses in different brain areas [18, 24, 26, 27, 154, 169]. No AC deficits as a consequence of disturbed AC differentiation or maturation [9, 175–178].
Cell pathological process leading to cell loss	Atrophy (?) Degeneration (?) Necroptosis (?) [33, 81, 86, 89, 92]	Apoptosis? Phagocytosis by MCs? [33, 93]	Atrophy [18, 88, 169]
Influence of medication	No significant influence of haloperidol on OL death in monkeys [97]. However, treatment with haloperidol and clozapine causes chemical alterations in human cultured OLs [94, 95]. Quetiapine enhances OL regeneration and myelin repair after cuprizone-induced demyelination [98].	Antipsychotics promote the differentiation of OPCs by regulating the transcription factors OLIG 1 and OLIG2 [118]. Olanzapine increased the numbers of new-born cells differentiating to the OL lineage but not the neuronal lineage [123]. Olanzapine stimulates proliferation but inhibits differentiation of cultured rat OPC [124]. Haloperidol activates quiescent OPCs in the adult mouse brain [125]. Quetiapine modulates OPC cell cycle and thereby facilitates OL differentiation [126, 127]. Haloperidol and clozapine have protective effects on energy-deprived OLN-93 OLs [128].	Haloperidol induces AC death in monkeys and cytotoxicity in human ACs in culture [97, 179, 180].
Putative impact on pathophysiology	Contributes to myelin loss [11, 46, 52, 54], Impairment of higher brain functions (including cognition) [37, 99], Influence on glutamatergic and dopaminergic neurotransmission [100–102].	Might significantly contribute to myelin loss. Possible influence on interneuron migration. Impairment of higher brain functions (including cognition).	Might contribute to disturbed neocortical neuron-glia interaction [18, 37, 99].
Possible therapeutic options in addition to antipsychotics	Antidepressants [188], Minocycline [201–203], Resveratrol [180], Aerobic exercise alone or in combination with clemastine [37, 39, 211], Engrafting of healthy cells [212]?	D-aspartate [199], Aerobic exercise [37, 39, 211], Engrafting of healthy cells [212]?	Antidepressants [188], Resveratrol [180], Aerobic exercise [37, 39, 211], Engrafting of healthy cells [212]?

## ASTROCYTES (ACs)

### Astrogliopathy is important in SCZ pathology

ACs are the second most common glial cell type in the human brain. These cells are heterogeneous with regards to morphology, physiological properties and spatial organisation [24, 132]. ACs are known to change with age in properties such as morphology, functions, inflammatory responses, interactions with neurons, molecular profiles, and regional variability [133]. Being associated through intercellular channels termed connexins, ACs form a functional reticular network in the CNS known as the AC syncytium [134]. ACs play multiple roles in normal brain function, including supplying neurons and OLs with substrates for energy metabolism, control of extracellular water and electrolyte homeostasis, expression and release of “gliotransmitters” and neuromodulators, recycling of aminoacidergic neurotransmitters via the glutamate/GABA-glutamine cycle at the tripartite synapse, modulation of immune and inflammatory responses, synthesis of multiple trophic factors, contribution to mitochondria biogenesis, regulation of the blood-brain barrier, and promotion of synapse formation and elimination [135]. Since almost all of these processes are abnormal in SCZ [87, 136–147], functional impaiments in ACs have been implicated in SCZ pathology.

Using GWAS, Goudriaan et al. found 6 AC gene sets associated with SCZ [58]. Among the many identified genes were glial fibrillary acidic protein (*GFAP*), apolipoprotein E (*APOE*), and solute carrier family 1 A2 and A3 (*SLC1A2* and *SLC1A3*) [58]. A number of studies have reported altered expression of mRNAs and encoded proteins in ACs of SCZ patients, although some of these findings have been contradictory, including those regarding expression of GFAP protein and mRNA [148–152], glutaminase and glutamine synthetase levels [153–155], abundance of enzymes and metabolites of the kynurenine pathway [140, 156], levels of the excitatory amino acid transporter proteins EAAT1 and EAAT2 [157], S100B abundance [67, 158], levels of ectonucleoside triphosphate diphosphohydrolase-1 and 2 mRNA [159], and number of DISC1 expressing ACs [160]. With regard to structural AC alterations, light microscopic studies have revealed swollen cathepsin-immunoreactive cortical ACs [161] and “gemistocytic” (i.e., swollen, reactive) GFAP positive ACs in SCZ brains [25, 26].

At the electron microscopic level, both dystrophic and swollen ACs were described by Uranova and co-workers [162, 163]. Another study found a significantly reduced number of mitochondria in hippocampal ACs in a subgroup of SCZ patients [164]. These structural alterations progressed with duration of illness [164]. Thus, astrogliopathy in SCZ may manifest itself in opposing morpho-functional changes, including atrophy with functional asthenia, and hypertrophy with elevated reactivity [86, 87, 89, 146, 151]. Hypertrophic ACs are thought to stand out by increased expression of GFAP, a hallmark of reactivity [146]. However, recent findings have shown that atrophic human brain ACs may also display increased GFAP expression [165], thus calling into question the usefulness of this cell marker to differentiate between atrophic and hypertrophic ACs. Whether or not hypertrophic ACs may pass into the state of atrophy has not been explored.

### Astrocyte deficits in schizophrenia

We suggest that the best way to count ACs to improve sensitivity is using Cresyl violet (Nissl)-stained sections. Although this approach is methodically demanding and some expertise is needed to differentiate between ACs and OLs [166], it delivers reliable results [19–22]. Nissl staining should be preferred over the use of cell markers since the latter may show variable expression levels against a background of unchanged AC cell densities in certain conditions. Applying Nissl staining, normal densities of dentate gyrus ACs were found in SZ [20], while a significantly decreased density of DISC1 immunoreactive ACs was found in this brain region in cases of the same cohort [160]. Using Nissl stained sections, Pakkenberg observed a significant decrease of glial cell densities in nucleus mediodorsalis thalami and nucleus accumbens [6], and Benes et al. saw a tendency towards lower glial cell densities in several neocortical areas in SCZ [167]. Unfortunately, both groups did not differentiate between ACs and OLs. Along similar lines, no alterations in AC densities were found in the neocortex, hippocampus and dentate gyrus of SCZ subjects after Nissl staining [19–22, 168].

Research carried out with the AC marker GFAP have yielded inconsistent results. A number of papers reported significantly decreased densities of GFAP-immunolabelled AC profiles in various cortical and subcortical brain regions in SCZ and in schizophrenia suicide completers [18, 24, 26, 27, 154, 169], while other studies found either no difference [164, 170, 171] or increased densities of GFAP immunopositive cells [149, 151, 172, 173]. From these contradictory data, illness-associated AC losses cannot be a widespread phenomenon. Nonetheless, reduced GFAP cell densities as reported by 6 independent research groups might speak for subtle, regionally circumscribed AC losses in a subset of SCZ cases, assuming that the observed reductions actually reflect cell losses [18, 24, 26, 27, 154, 169]. However, it should be noted that cell density measurements may be biased due to tissue shrinkage after fixation and staining. Additionally, changes in cell density can occur without any change in the actual number of cells, which may indicate a functional impairment.

The above findings raise several questions: (i) which AC subpopulations might be affected? (ii) is there an influence of antipsychotic treatment on AC demise? and (iii) how important are AC losses as part of a wide-spread astrogliopathy? Concerning the first question, it is likely that a small portion of compromised ACs die by atrophy and not by degeneration [86, 88, 169]. Since some ACs are GFAP-negative [174], it cannot be said with certainty from classical histopathological approaches, which subtype of ACs is most affected. As atrophy is a reversible process, there may exist ways to rescue these cells. Importantly, although several lines of evidence suggest dysregulated AC differentiation in childhood onset and adult onset SCZ [9, 175–178], there is no evidence of cell loss during the disturbed AC maturation process (in contrast to OL maturation in SCZ; see above). However, definitive proof of the absence of AC progenitor deficits is still lacking, since there are still no quantitative studies using appropriate markers for these cells.

Investigations on the possible influence of antipsychotic treatment on AC cell densities have mostly dealt with post-mortem brains of chronic SCZ patients who had received antipsychotic medication over varying numbers of years. Therefore, it cannot be ruled out that AC losses are a consequence of long-term medication. In support of this assumption are findings of haloperidol-induced cytotoxicity in human ACs [179, 180], reduction of AC (but not OL) densities in macaque monkey brains after chronic exposure to antipsychotics [97], and effects of chronic antipsychotic medication on AC marker expression in rat brains [181]. However, recent MRI studies have demonstrated significantly reduced concentrations of myo-inositol in the prefrontal cortex at an early, state of acute SCZ, prior to medication. This suggests that dysregulation of ACs may be an initial, treatment-independent event [182]. Whether or not long-term treatment is involved in the worsening of the functional situation of compromised ACs, remains an open question.

One question that remains is whether AC losses affect SCZ pathology? Rats injected with the AC specific toxin L-*α*-aminoadipate in the medial prefrontal cortex showed attentional set-shifting and a decline of learning and working memory [183]. However, histological analysis of rat brain sections revealed massive AC loss in the targeted region as opposed to a slight to moderate decrease found in SCZ. In view of the high impact of functionally compromised ACs on the pathophysiology of SCZ, it is conceivable that the absence of a small number of ACs has only a minimal influence [184]. However, when AC losses appear at “strategic places” (for example, a 32% reduction in the GFAP-area fraction in layer V of the dorsolateral prefrontal cortex [18]), they could disrupt neuron-glia interactions in this layer, and thus have a dysfunctional effect on prefronto-striatal circuits in SCZ [18]. Notably, these late maturating circuits are probably more vulnerable towards glial hypofunction at the typical adolescent or young adult age of onset of SCZ [4].

A summary of the key OL and AC biomarkers identified in SCZ is given in Table 3.

**Table 3:** Summary of the key OL and AC biomarkers identified in schizophrenia.

Glial cell type	Markers	Description/Definition	Finding in schizophrenia
Oligodendrocytes (OLs)	MAG (Myelin-Associated Glycoprotein)	Protein associated with myelin and the maintenance of myelinated axons.	Decreased expression observed, contributing to impaired myelination [56, 57].
	CNPase (2’,3’-Cyclic-Nucleotide 3’-Phosphodiesterase)	Enzyme involved in myelin production and maintenance.	Reduced expression, implicating deficits in white matter integrity [57].
	OLIG2 (Oligodendrocyte Transcription Factor 2)	Transcription factor critical for oligodendrocyte differentiation and myelination.	Decreased expression linked to impaired oligodendrocyte maturation [35, 82].
	ADAM12	Metalloprotease involved in cell-cell and cell-matrix interactions, implicated in oligodendrocyte development.	Reduced density in the anterior cingulate white matter of schizophrenia patients [64].
Oligodendrocytes (OLs) and Astrocytes (ACs)	S100B	Calcium-binding protein in astrocytes, involved in cellular functions like energy metabolism.	Increased levels in brain and serum, often linked to glial activation [67, 158].
Astrocytes (ACs)	GFAP (Glial Fibrillary Acidic Protein)	Cytoskeletal protein expressed in mature astrocytes.	Altered GFAP expression with studies showing both increased and decreased levels in different brain regions [148, 150–152, 154].
	Glutamine Synthetase (GS)	Enzyme that catalyzes glutamine synthesis from glutamate and ammonia.	Elevated expression in some regions (e.g., thalamus), affecting glutamate regulation [153, 155].
	DISC1	Disrupted-in-Schizophrenia 1, a protein involved in neurodevelopment and synaptic function.	Decreased density of DISC1-positive astrocytes in the dentate gyrus [160].
	Glutaminase	Enzyme that converts glutamine to glutamate, crucial for excitatory neurotransmission.	Increased expression of glutaminase mRNA in the thalamus of schizophrenia patients [153].
	D-Amino Acid Oxidase	Enzyme that degrades D-serine and other D-amino acids.	Increased expression of D-amino acid oxidase in the hippocampus of schizophrenia patients [138].

## Therapeutic opportunities

A comparison of key histological features, clinical implications and mechanistic insights between SCZ and neurodegenerative diseases is given in Table 4. These differences suggest that therapeutic interventions which help to rescue glial cell deficits may be a viable approach as novel SCZ treatments.

**Table 4:** Comparison of key features, clinical implications and mechanistic insights between schizophrenia and selected neurodegenerative diseases.

Feature	Schizophrenia	Alzheimer’s disease	Parkinson’s disease	Frontotemporal dementia
Primary affected cell type	Glial cells (atrophy, degeneration or impaired regeneration and maturation)	Neurons	Neurons	Neurons
Histological features: neurons	No protein deposits or inclusions in neurons Some neuronal abnormalities: smaller cell somata and fewer synapses (likely secondary due to oligo- and astroglial deficits in the support of neurons, see Table 1) Very rare neuronal cell loss, if any	Neurofibrillary tangles in neurons Amyloid plaques in the extracellular space Reduced neuron numbers (consequence of cell death due to protein aggregates) with hippocampal / temporoparietal focus [213].	Inclusions comprised of α-synuclein (α-syn), i.e. Lewy bodies and Lewy neurites Reduced dopaminergic neuron numbers (consequence of cell death due to protein aggregates) in the substantia nigra [214].	Tau and TAR DNA-binding protein 43 (TDP-43) inclusions in neurons Reduced neuron numbers (consequence of cell death due to protein aggregates) Spongiform degeneration of the superficial laminae of the cortex [215, 216].
Histological features: glia	Reduced oligodendrocyte numbers and myelination Reduced astrocyte densities Dysregulated microglial activation?	Secondary astrogliosis^a^ & Activated microglia^a^ =reaction to amyloid plaques, neuronal damage or death [213].	Secondary astrogliosis^a^ & Activated microglia^a^ =reaction to alpha-synuclein deposits, neuronal damage or death [214].	Secondary astrogliosis^a^ & Activated microglia^a^ =reaction to Tau and TDP-43 deposits, neuronal damage or death [215, 216].
Leading clinical symptomss	Positive Symptoms: Hallucinations (most commonly auditory), delusions (fixed false beliefs), and disorganized thinking/speech. Negative Symptoms: Reduced emotional expression (flat affect), lack of motivation (avolition), and social withdrawal. Cognitive Symptoms: Impaired attention, memory, and executive function. Disease course: relapsing-remitting, with or without progressive decline. Potential progressive decline in later stages	Memory loss and disorientation. Problems with speech and writing / aphasia: Beginning with word-finding difficulties, reduced vocabulary. Visuospatial problems: Difficulty understanding visual information, visoconstruction (drawing), spatial orientation. Behavioural changes and hallucinations in later stages [216]. Disease course: chronic-progressive [217].	Motor symptoms: tremor, rigidity, akinesia. Motor disability in later stages. Depression Bradyphrenia (slowness of thought) Cognitive impairment in later stage, hallucinations in overlapping Lewy body dementia or as dopaminergic treatment side effect [218]. Disease course: chronic-progressive.	Changes in personality, social behaviour and conduct: disinhibition, apathy, and loss of empathy. Executive Dysfunction: Impairments in planning, judgment, and problem-solving. Problems with speech: progressive aphasia [219]. Disease course: chronic-progressive.

^a^This involves the activation and proliferation of astrocytes and micoglia in an attempt to mitigate the damage and to restore homeostasis in the brain environment.

### Effects of psychiatric medications on OLs, OPCs or ACs

First- and second-generation antipsychotics have been shown to modulate OLs [94] and ACs [181, 185]. Some animal and cell culture studies found *detrimental effects* of chronic treatment on OLs, OPCs or ACs, which resulted in reduced cell count or function [96, 97, 124, 180]. On the other hand, there have been indications for *glioprotective effects*, particularly in the cases of quetiapine, olanzapine and clozapine, on the proliferation of OPCs, myelin repair and maturation of OLs [95, 98, 118, 123–128, 180].

AC express receptors for neurotransmitters such as dopamine, GABA, glutamate and serotonin, which are targets for antipsychotics [186]. Additionally, ACs produce the enzyme glutathione synthase and are pivotal in the synthesis and regulation of glutathione, a critical antioxidant involved in reducing oxidative stress; they are capable of modulating inflammation pathways, mitochondrial function, and glutamate metabolism [180].

Some studies have indicated that antipsychotics may also have protective effects on cells through induction of neurotrophic factors. Shao et al. tested both atypical (quetiapine and clozapine) and typical (haloperidol) antipsychotics for their ability to induce secretion of the glial cell line–derived neurotrophic factor (GDNF) from C6 glioma cells [187]. They found that all three drugs induced GDNF release from an unchanged number of cells.

After analysing findings from several post-mortem studies on depressed subjects Banasr and colleagues concluded that structural alterations in depression result from atrophy and loss of neurons and glial cells that can be blocked or even reversed by antidepressant treatment [188]. Since symptoms of depression are common in SCZ, many patients receive antidepressants in addition to antipsychotics [189]. Unfortunately, there are no studies which compare glial cells losses in SCZ patients with and without antidepressant treatment.

Taken together, these findings suggest that glioprotection is likely to be an additional mechanism of action to the known modulation of synaptic neurotransmission for some antipsychotics. Due to lack of information about glial cell deficits in brains of untreated SCZ patients, it is difficult to say whether or not medication helps to mitigate glial abnormalities or even prevent glial cell losses. The availability of literature on post-mortem SCZ brains and glia from before 1950, when chlorpromazine was discovered, could help to resolve this issue. However, the substantiated finding that, despite antipsychotic treatment, SCZ patients may suffer from deficits of OLs, OPCs and ACs is a clear indication that antipsychotic medication is not enough to fully prevent glial cell losses.

### Stem cell research and cell therapy

As mentioned above, OLs and ACs probably die by pathological processes which are basically reversible. Hence, compounds which target these pathways should be investigated for successful *glioprotection*. Of potential relevance to SCZ, a recent review focused on use of induced pluripotent stem cells (iPSCs) to increase our understanding of the involvement of ACs, MCs and OLs in the pathology of neurodegenerative diseases [190]. The studies involve using these cells in the production of cellular and organoid cultures, as well as intracranial transplantation to potentially provide useful disease models and insights into new drug targets and therapies. iPSCs can self-renew and differentiate into a variety of cells. These can be used to replace damaged or dead cells in animal models of various diseases and there has also been some success in human studies. In a patient with idiopathic Parkinson’s disease, iPSCs derived from the patient were differentiated into midbrain dopaminergic progenitor cells and implanted into the putamen, which led to improvement in symptoms over 18-24 months [191]. Although the original focus of research was on generation of neuronal cells, iPSCs can also be differentiated into glial cells and thus represent a potential future treatment strategy for SCZ. It is possible that iPSCs could also be used to implant functional OLs leading to an improvement in connectivity in SCZ.

### Remyelinating agents

Studies on the promotion of remyelination in diseases such as multiple sclerosis could also be applicable in SCZ. Münzel, and Williams, reviewed existing drugs that promote remyelination by OLs to enhance white matter and brain connectivity [192]. Clinical trials are either planned or underway for this purpose with the testing of compounds such as alemtuzumab, dimethyl fumarate, LINGO-1 antibodies, and IgM monoclonal antibody 22. Other potential remyelinating compounds described include clemastine, GSK239512, opicinumab, GNbAC1, simvastatin, biotin, quetiapine, fumarate, and domperidone. LINGO-1 is a negative regulator of OPC differentiation and remyelination [193], and reagents which block this pathway could improve OPC differentiation efficiency. IgM22 could also have a positive effect on white matter integrity in SCZ through its association with anti-apoptotic signalling in pre-myelinating OLs [194]. Notch-1 is also a negative regulator of remyelination [195] and Wnt pathway signalling appears to delay oligodentrocyte maturation [196]. In addition, the application of sonic hedgehog (Shh) and a retinoid X receptor (RXR) agonist resulted in increased OPC proliferation and differentiation in animal experiments [197, 198]. Finally, D-aspartate administration was shown to stimulate OPC maturation and accelerate myelin recovery during remyelination, which improved motor coordination [199].

### Anti-inflammatory agents

Compounds that possess anti-inflammatory properties, such as nonsteroidal anti-inflammatory drugs (NSAIDs), glucocorticoids, and minocycline, have been investigated for their potential use in neuropsychiatric disorders [200]. Use of minocycline as an adjunctive treatment in SCZ is thought to have an overall beneficial effect [201], which may include protective roles for glial cells. In cell culture minocycline was found to reduce OL damage caused by MC activation [202] and to protect OPCs against injury caused by oxygen-glucose deprivation [203].

### Antioxidant drugs and natural products

Numerous studies have found increased oxidative stress and redox imbalance in SCZ, caused by both genetic predisposition and environmental influences, such as immune dysfunction. In addition to neuronal damage caused by oxidative stress, damage to OLs can also occur. OLs are vulnerable to oxidative stress and glutathione deficiency can lead to arrest of OPC proliferation and apoptosis, resulting in hypomyelination. In addition, death of OLs or OPCs can also increase neuroinflammation and oxidative stress. On the other hand, reduction of oxidative stress could lead to an improvement of white matter integrity in SCZ patients. The effectiveness of some antioxidant drugs and natural substances has already been investigated [204], including N-acetylcysteine (NAC), ginkgo biloba, selegiline, allopurinol, polyunsaturated fatty acids (PUFAs) and vitamins E and C. In a meta-analysis, administration of the antioxidant and free radical-scavenging NAC led to significant improvement in psychotic symptoms and cognition after 24 weeks [205]. In addition, substances that act on redox-regulated transcription factors have also been proposed. For example, application of the Nrf2 transcription factor activator sulforaphane led to an increase in blood and brain glutathione (GSH) levels in healthy adults which could lead to improved cognition in patients with SCZ [206, 207]. Since typical antipsychotics such as haloperidol presumably lead to increased oxidative stress in ACs, NAC has been proposed as adjunctive treatment [180, 208].

Schmitz et al. emphasized the potential of natural substances such as curcumin, isoflavones, and sulforaphane, endogenous mammalian compounds including lipoic acid and guanosine (a guanine-based purine) and resveratrol, which supposedly have glioprotective effects [180]. Their protective effects on glial cells include anti-inflammatory properties partly mediated by inhibition of MC activation, antioxidant effects with improvement of mitochondrial function, influence on glutamate metabolism and survival and differentiation of OLs [209]. Relevant metabolic pathways that could be targeted include NFκB inhibition and Nrf2/HO-1 activation. Resveratrol may influence various AC functions such as inflammatory response, glutamate homeostasis, and antioxidant defense and trophic factor release. In addition, this natural compound may have glioprotective effects on OLs and MCs. There are initial clinical experiences with resveratrol add-on therapy in the treatment of negative symptoms in patients with stable SCZ [210].

### Physical exercise

A simple and potentially effective way to positively influence the fate of glial cells in SCZ is long-term physical activity [37, 39]. The rationale for this comes from studies which found that OLs in developing and adult rodent brains are highly responsive to physical exercise. For example, NG2-expressing cell proliferation increases in exercising juvenile rats [211]. Importantly, there is evidence that *aerobic exercise* has an impact on glial cells in humans. Papiol et al. [39] and Falkai et al. [37] investigated SCZ patients and healthy controls who performed aerobic exercise, as well as patients who only played table soccer. MRI-based assessments were carried out at baseline and after 3 months. It was found that the polygenic burden associated with OPCs and radial glia significantly influenced the volume changes between baseline and 3 months in hippocampus subfields in SCZ patients performing aerobic exercise. Also, aerobic exercise training led to a volume increase in the hippocampal subfield CA4, together with improved cognition in individuals with SCZ [39]. Analysis of cell-type specific SCZ polygenic risk scores showed that exercise-induced volume increase significantly correlated with OPCs. Moreover, higher OPC-or radial glia-associated genetic risk burden was associated with a smaller volume increase, or even a decrease, during exercise. The authors of both communications propose that aerobic exercise training combined with the histamine blocker clemastine or other pro-myelinating drugs might help to promote regeneration of myelin plasticity in SCZ. However, further studies are required to determine whether or not physical activity of SCZ patients prevents glial cell death.

## Conclusions and future perspectives

In this review, we have highlighted the point that macro-glial networks are disrupted in SCZ, distinct from the changes seen in neurodegenerative conditions which appear to arise mainly from neuronal deficits (Table 4). We described two different ways that might lead to drastically reduced OL numbers in SCZ: (i) death of mature, myelinating OLs by atrophy and/or degeneration; and (ii) deficits arising from failure of OPCs to complete their development into mature OLs. In contrast, minor AC deficits can be found in SCZ and these cells are thought to die by atrophy. Glial cell deficits in SCZ bring about many serious functional consequences, with myelination deficits and, thereby, disrupted neuronal connectivity being an obvious critical outcome. Since antipsychotic treatment alone is not able to prevent glial cell losses, there is urgent need to identify new therapeutic options that effectively target these pathways. Given the distinction from neurodegenerative disorders, the proposed approaches include the application of antidepressants and other chemical compounds, remyelinating agents, natural products, physical exercise and engrafting healthy glial cells into brains of SCZ patients. A better understanding of gliopathologies in SCZ could lead to development of additional tools in the investigation of this psychiatric illness, with a focus on diagnostics, new treatments and outcome assessments.
